# Remarks on Mr. Duck's Case of Crural Hernia

**Published:** 1808-07

**Authors:** 


### *To the Editors of the Medical and Physical Journal.*

Gentlemen,

I Beg leave to offer a few remarks on Mr. Duck's case of Crural Hernia, recorded in your last Journal, as I am led, by his statement, to consider it in a different point of view from the impression it appears to have made on *him.*

It surely may be considered as very extraordinary, that five inches of the intestinal canal should have been thrown off in a sphacelated state, and yet a proper discharge by stool should have been procured only *on the day before* its separation, and that it should not, *afterwards,* have entirely interrupted the natural evacuation of the fæces for a longer period than is described by many days.

The

The case is said to have been a strangulated crural hernia on the right side, accompanied with considerable inflammation of the protruded intestine, and also of the integuments. It was treated in the usual manner, and appears to have returned into the abdomen, or been relieved from stricture, after the injection of a tobacco clyster; as we are informed, that the patient had in consequence of it *too copious evacuations* by stool, and became easy. She is said to have continued also from this time free from pain in the bowels, and to have had afterwards, by means of an opening medicine, *another proper discharge by stool.* Nothing can be more decisive of the cessation of the stricture. The inflammation, however, it is said, terminated in gangrene; that five inches of the intestine sloughed off, and that the natural discharge by stool, after only some *days* interruption, began gradually to return, and at last was completely restored.

I think with Mr. Duck, it is highly probable that this case is similar to that of Abraham Pike, which he next proceeds to quote from the 3d Vol. of the Medical Observations and Inquiries: but I think so, on very different grounds from his. And that Mr. Duck should quote this case without *the explanation,* and publish it in this separated manner, as an instance of complete spontaneous recovery from a sphacelation of the entire substance of the intestinal canal, and therefore, as a similar occurrence to the one he superintended, is truly extraordinary; since the case he has quoted, is published in the place to which he refers his reader, *purposely* to shew what had been *considered as a mortified intestine,* was a mortification only of the *appendicula ceci vermiformis.*

The circumstances were shortly these: Abraham Pike was operated on for a strangulated hernia, and six inches of what was *imagined* to be the whole annular substance of the intestine, were cut off in a sphacelated state, and that after a short interruption only, the fæces resumed their natural passage through the bowels, and the patient lived many years in good health. This case was published at the time in the Medical Essays, and attracted a considerable degree of attention.

Some time afterwards, when Pike died, his body was opened by Mr. Simmons of Exeter, in the presence of several medical gentlemen, for the purpose of examining in what manner so singular an occurrence had taken place, when it was clearly ascertained that the part which had been been separated, and which had been supposed to be *intestine*, was only the *appendicula vermiformis*.

Mr. Simmons then, published this satisfactory explanation in the 3rd. Volume of the Medical Observations and Inquiries, adding the substance of the case itself, as it appeared in the Medical Essays, by way of note. It is this *note only*, which Mr. Duck has now quoted in support of his opinion, without noticing Mr. Simmons's explanation, to which it is subjoined, and detached from which, it is manifestly incomplete, and calculated to mislead the judgment.

Mr. Duck's case may or may not have been as he imagined; but I trust I have shewn you that the authority he has quoted, *if taken entire*, is decidedly against his opinion, and it is probable if he should have an opportunity of examining the parts after death, he may be able to explain it in the same satisfactory manner as was done in that instance.

Numerous cases have occurred in strangulated hernia, where, after portions of *the side* of the intestine have sloughed off, the fæces, in course of time, have been discharged through their natural passage, in consequence of an adhesion formed between the remaining part and the peritonæum; but I believe scarcely an instance has been properly authenticated, where the *whole substance* has been so separated for five or six inches, and without any care being taken to unite the living portions to each other, that a cure has been spontaneously effected.

I am, &c.

T. H.

*June*, 9, 1808.

